# Efficacy of Conservative Interventions for Musculoskeletal Conditions on Pain and Disability in Active Serving Military Personnel—A Systematic Review

**DOI:** 10.1093/milmed/usac409

**Published:** 2023-01-31

**Authors:** Courtney L Bounds, Michel W Coppieters, Hayley W Thomson, Brianna Larsen, Kerrie Evans

**Affiliations:** Menzies Health Institute Queensland, Griffith University, Brisbane (Nathan), QLD 4111, Australia; Menzies Health Institute Queensland, Griffith University, Brisbane (Nathan), QLD 4111, Australia; Amsterdam Movement Sciences—Program Musculoskeletal Health, Faculty of Behavioural and Movement Sciences, Vrije Universiteit Amsterdam, Amsterdam 1081 BT, The Netherlands; Menzies Health Institute Queensland, Griffith University, Brisbane (Nathan), QLD 4111, Australia; Gold Coast University Hospital, Gold Coast Hospital and Health Service, Southport, QLD 4125, Australia; Griffith Sports Physiology and Performance, Griffith University, Southport QLD 4222, Australia; Healthia Limited, Brisbane, QLD 4006, Australia; School of Health and Medical Sciences, University of Southern Queensland, Ipswich, QLD 4305, Australia; Faculty of Medicine and Health, The University of Sydney, Sydney, NSW 2006, Australia; Griffith Sports Physiology and Performance, Griffith University, Southport QLD 4222, Australia; Healthia Limited, Brisbane, QLD 4006, Australia

## Abstract

**Introduction:**

Musculoskeletal (MSK) injuries and associated pain disorders are one of the leading causes for soldiers not being medically fit for deployment, impacting force capability and readiness. Musculoskeletal pain continues to be a leading cause of disability within military services and is associated with a substantial financial burden. A better understanding of the effectiveness of MSK pain management strategies is required. This review was designed to determine the efficacy of nonsurgical interventions, such as physiotherapy, exercise, pharmacology, and multidisciplinary programs, to manage MSK conditions in active serving military populations.

**Materials and Methods:**

MEDLINE, Embase, CINAHL, and SPORTDiscus were searched to identify relevant randomized clinical trials. Recommended methods were used for article identification, selection, and data extraction. The Cochrane Risk of Bias tool and the Grade of Recommendation, Assessment, Development, and Evaluation were used to appraise the studies. Where possible, meta-analyses were performed. The review was conducted according to the PRISMA guidelines.

**Results:**

Nineteen articles (1,408 participants) met the eligibility criteria. Low back pain (LBP) was the most frequently investigated condition, followed by knee pain, neck pain, and shoulder pain. Early physiotherapy, exercise and adjunct chiropractic manipulation (for LBP), and multidisciplinary pain programs (physiotherapy, occupational therapy, and psychology) (for chronic MSK pain) improved pain (standardized mean difference ranged from −0.39 to −1.34; low strength of evidence). Participation in multidisciplinary pain programs, adjunct chiropractic manipulation, and early physiotherapy improved disability (for LBP) (standardized mean difference ranged from −0.45 to −0.86; low to very low strength of evidence). No studies evaluated pain medication. Dietary supplements (glucosamine, chondroitin sulfate, and manganese ascorbate), electrotherapy, isolated lumbar muscle exercises, home cervical traction, or training in virtual reality showed no benefit. The studies had a high risk of bias, were typically underpowered, and demonstrated high clinical heterogeneity.

**Conclusions:**

Currently available randomized clinical trials do not provide sufficient evidence to guide military organizations or health care professionals in making appropriate treatment decisions to manage MSK pain in active serving military personnel. Future research is essential to enable evidence-based recommendations for the effective management of MSK pain conditions in this unique population.

## INTRODUCTION

Pain and musculoskeletal (MSK) conditions limit human performance.^1^ Both are prevalent in active individuals,^1^ including active serving military personnel.^2,3^ The physical demands of military-specific occupations can be unpredictable. Military personnel can be exposed to high operational tempos and variable environmental conditions.^4^ These factors, combined with significant training loads and high-performance requirements, increase the risk of MSK injury or experiencing pain.^5^

Musculoskeletal injuries and associated pain disorders are one of the primary causes for soldiers not being medically fit to be deployed and are the leading cause for disability within military services.^6^ In the U.S. Army, more than 50% of soldiers sought medical care for MSK injuries in 2018,^2^ resulting in over 2 million medical encounters,^2^ more than 8 million limited duty days,^7^ and US$434 million in direct patient care costs.^7^ Similar findings have been reported in other military organizations. For example, the incidence of MSK injury in The Netherlands Armed Forces ranged from 12.5 to 53.3 per 100 person-years across units.^8^

Musculoskeletal-related pain presentations are the most common primary pain diagnoses in U.S. soldiers.^9^ Approximately one in three (34.7%) U.S. military personnel experienced low back pain (LBP) between 2017 and 2018.^10^ Furthermore, back and neck pain are reportedly more common to become persistent or chronic in nature (i.e., beyond the expected tissue healing time, often defined as a duration of >3 months) compared to other pain conditions.^11^ The prevalence of chronic pain is greater than 40% in serving military personnel returning from deployment.^5,12^ Persistent pain can increase an individual’s risk of developing secondary health deficits including psychological complications and disability.^5,7^ Furthermore, poorly managed persistent pain has been identified as an independent risk factor for suicidal ideation and behavior in veterans.^13^

There is a concern that the true burden of MSK injuries on military health systems is not adequately represented in the literature. Injury concealment is common, with more than half of surveyed soldiers within a combat brigade stating that they had an injury and they did not report to medical services.^14^ Consequently, MSK injury and pain across military populations are likely to be underreported^14^ and present a significantly larger challenge to the efficiency and operational effectiveness of military organizations.^15^

Currently, there are several reviews for the management of MSK pain in general^16,17^ and athletic populations.^18^ However, the findings of these reviews may not be applicable to active military populations. Considerations need to be made for military occupational tasks, such as the use of weapon systems or operating heavy machinery, both of which are fundamental in combat environments.^19^ For example, certain medications have side effects on cognitive function and mood^20^ and are therefore not compatible with performing full military duties. Another consideration in military medicine is the use of over-the-counter medications, such as nonsteroidal anti-inflammatory drugs (NSAIDs). The majority (81%) of soldiers reportedly use over-the-counter medications as an injury management strategy.^14^ Prescription of NSAIDs is associated with up to five times greater risk of stress fractures in military training.^21^ Thus, persistent use of over-the-counter medications may potentially exacerbate the overarching issue of MSK pain and reduced force capability.

The purpose of this review was to summarize the current evidence for the efficacy of nonsurgical interventions for MSK conditions within the unique structure of military medicine. The review is limited to active serving populations, as veterans and recruits differ from active serving populations in many ways (e.g., work stressors, exposure to training, and deployment environments), and thus the outcomes of interventions may differ.

## METHODS

The study is reported according to the PRISMA statement^22^ and the Measurement Tool for Assessing Systematic Reviews.^23^

### Eligibility Criteria

Studies were considered for inclusion in this review if they met the following inclusion criteria: (1) study design: randomized clinical trial (RCT); (2) participants: active serving military personnel only; (3) pathology: acute or chronic MSK conditions; (4) treatment: conservative (i.e., nonsurgical) interventions, such as physiotherapy, exercise, pharmacology, and multidisciplinary programs; and (5) outcome measures: pain intensity, disability, and/or global perceived effect. Studies were excluded if (1) the study group(s) were not active serving military personnel, i.e., veterans, trainees, recruits, or beneficiaries (e.g., dependents of military personnel); (2) surgery was part of a multidisciplinary program; or (3) the intervention was postoperative management.

### Outcome Measures

Studies that assessed at least one of the following self-reported outcome measures were considered: (1) a measure of severity or intensity of pain (e.g., visual analog scale or numerical pain rating scale); (2) a measure of disability (e.g., Oswestry Disability Index or Roland–Morris Disability Questionnaire), or (3) a measure of the overall treatment effect (e.g., Global Perceived Effect of Global Rating of Change score).

### Literature Search

MEDLINE, Embase, CINAHL, and SPORTDiscus were searched from their respective inception dates up to October 2021. The search strings were developed in collaboration with a university research librarian. Reference lists of included papers and military medicine conference abstracts from the past 10 years were screened for additional studies. Studies had to be published in English in peer-reviewed journals.

### Study Selection

Retrieved references were imported in Covidence (Veritas Health Innovation, Australia).^24^ Two investigators (C.L.B. and H.W.T.) independently screened the titles and abstracts for potential inclusion. When the title and abstract suggested that the study met the criteria, or when insufficient information was available in the title and abstract, the full-text article was screened. Discrepancies between the two investigators were resolved via discussion, or a third investigator (K.E.) would be consulted if the two investigators could not resolve the discrepancy.

### Data Extraction

Data from each study that met the selection criteria were extracted independently by two reviewers (C.L.B. and H.W.T.) using a standardized data collection form. Data extracted for each study included publication details, study design, study setting, sample size, demographical patient data, outcome measures, and summary statistics. If numerical data were not available, the corresponding author of the publication was contacted via email. If no response was received, a digital screen ruler (Adobe Acrobat software, Adobe Inc., San Jose, CA, USA) was used to extract data from the published graphs.

### Risk of Bias

Included studies were assessed independently for methodological quality by two reviewers (C.L.B. and H.W.T.) using the Cochrane Risk of Bias 2.0 tool.^25^ Domain-level judgments were made to provide the basis for an overall risk of bias judgment for each included study. Domains included bias arising from the randomization process, deviations from the intended interventions, missing outcome data, measurement of the outcome, and in selection of the reported results. Any discrepancies between reviewers were discussed and resolved. If a consensus was not reached, a third reviewer (K.E.) was consulted.

### Strength of the Evidence

The strength of the body of evidence was assessed using the Grade of Recommendation, Assessment, Development, and Evaluation (GRADE) approach.^26^ All included studies had to be RCTs and therefore allocated an *a priori* ranking of high. Domains that reduced the strength of the evidence included risk of bias, inconsistency of results, indirectness, and imprecision.

### Synthesis of Results

A quantitative synthesis (meta-analysis) using random effects modeling was performed if the degree of clinical diversity of the included studies was deemed acceptable and statistical heterogeneity was assessed as low (*I*^2^ < 40%).^27^ If a meta-analysis was not possible, a narrative synthesis was performed. Statistical analyses were performed using RevMan (Version 5.4) software.^28^ The standardized mean difference (SMD) was calculated where adequate data were available.

## RESULTS

**FIGURE 1.**
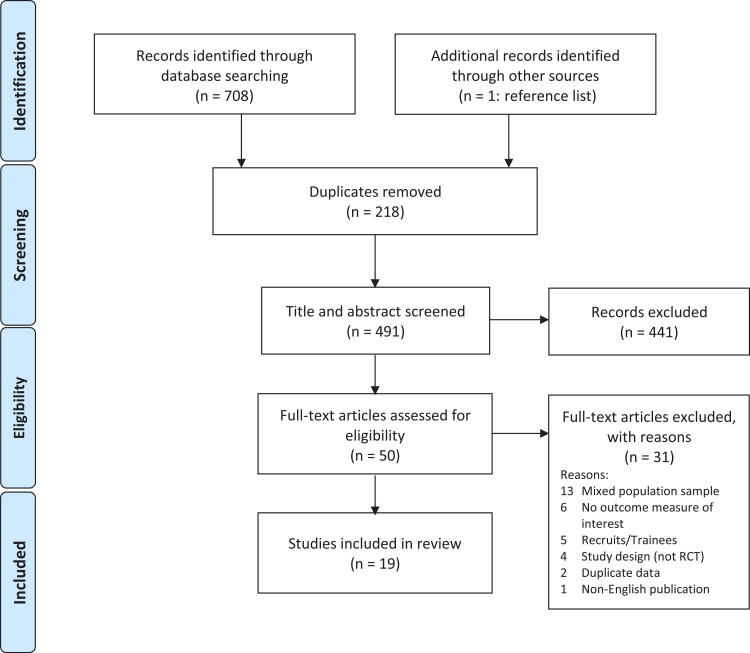
Overview of the selection of studies.

Electronic and manual searches identified 708 references. After removal of duplicates (*n* = 217), 491 titles and abstracts were screened for suitability. Of these, 50 were selected for full-text review. Thirty-one studies were excluded for not meeting the selection criteria, leaving a final yield of 19 articles for data extraction and analysis.^29–47^ Articles were most frequently excluded for recruiting mixed populations (*n* = 13), trainees or recruits (*n* = 5), or not using an outcome of interest (*n* = 6). A flowchart of this process is presented in Figure 1.

Meta-analyses could not be performed because of highly variable study characteristics in the included studies. Considering this variability, formal testing of heterogeneity was deemed unnecessary, and thus, quantitative pooling of results was deemed inappropriate.

### Characteristics of Included Studies

The 19 included studies were published between 1995 and 2021 and involved a total of 1,408 participants. Study characteristics and a summary of results are presented in Supplementary Table S1. Thirteen of the included studies involved the U.S. military services,^29,31–34,37–40,44–47^ and two studies were conducted in Dutch Army populations.^35,36^ Other services included the Canadian^41^ and Iranian Military^42^ and the Israeli^43^ and Danish Air Force.^30^ All branches of military were represented: three studies included only air force personnel,^30,40,43^ two studies included only navy personnel,^29,37^ three studies were based in army populations,^32,35,36^ and 11 studies included either all branches of service or did not specify otherwise.^31,33,34,38,39,41,42,44–47^ Five studies included male participants only,^30,35,37,40,42^ and 14 studies included both male and female personnel.^29,31–34,36,38,39,41,43–47^

Low back pain was the most frequently investigated MSK condition.^29,31,32,34–40,42,45,46^ Three studies addressed interventions for acute LBP.^31,32,34^ Two studies assessed knee pain,^44,47^ and one study included both knee pain and LBP.^37^ Other evaluated regions included neck pain^30,43^ and shoulder pain,^41^ and one study included any chronic MSK pain condition.^33^

The interventions in the experimental groups for the management of MSK pain included early physiotherapy intervention,^39^ multidisciplinary pain programs (physiotherapy, occupational therapy, and psychology),^30,33^ adjunct manipulative treatment,^31,34,46^ exercise therapies,^32,35,36,40–43,45,47^ a dietary supplement of combined glucosamine, chondroitin sulfate, and manganese ascorbate,^37^ and electrotherapy modalities.^38,44,45,47^ The interventions in the control groups consisted of usual care,^29,31,33,34,38,39,41,43,45,47^ wait-list,^30,35,46^ exercise,^40,42,44^ physiotherapy,^36^ and ice application.^32^

### Summary of Findings

**FIGURE 2.**
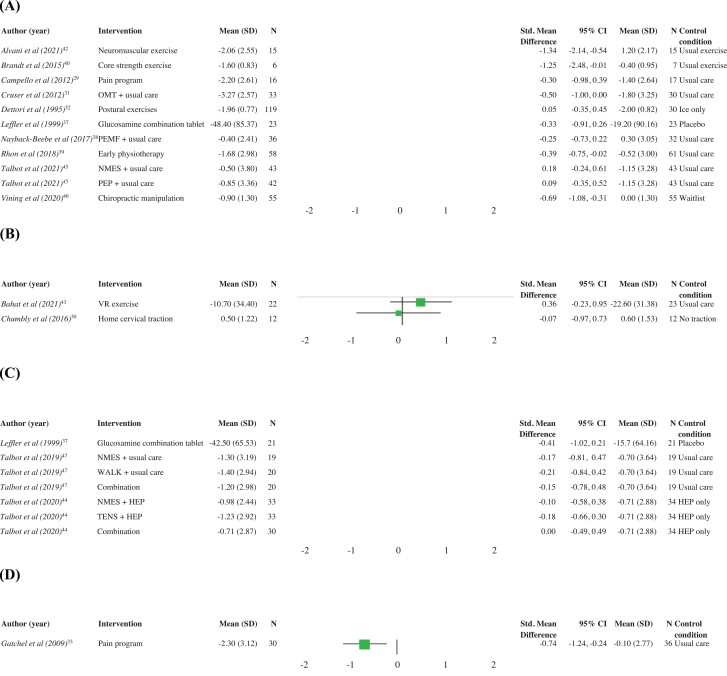
Pain intensity. (A) Interventions for low back pain. (B) Interventions for neck pain. (C) Interventions for knee pain. (D) Interventions for musculoskeletal pain. HEP: home exercise program; NMES: neuromuscular electrical stimulation; OMT: osteopathic manipulative therapy; PEMF: pulsed electromagnetic frequency; PEP: progressive exercise program; SMD: standardized mean difference; TENS: transcutaneous electrical nerve stimulation; VR: virtual reality; WALK: graduated strength walking program.

**FIGURE 3.**
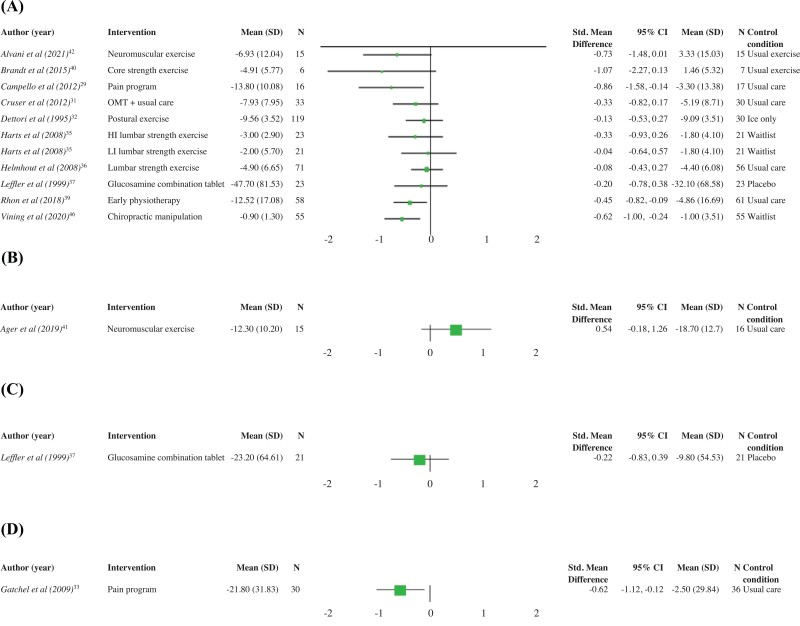
Pain disability. (A) Interventions for low back pain. (B) Interventions for shoulder pain. (C) Interventions for knee pain. (D) Interventions for musculoskeletal pain. HI: high intensity; LI: low intensity; OMT: osteopathic manipulative therapy; SMD: standardized mean difference.

Of the 19 included studies, 18 were suitable for quantitative analysis.^29–33,35–47^ Results of the statistical analysis are presented in Figures 2 and 3.

#### Low back pain

Exercise therapy for LBP revealed varied results. A study of 13 U.S. Air Force personnel revealed strength exercises for core muscles for LBP reduced in-flight pain severity (SMD: −1.25; 95% CI: −2.48 to −0.01) (Fig. 2A); however, pain disability scores were not different when compared to continuation of the participants’ usual exercise routine (SMD: −1.07; 95% CI: −2.27 to 0.13) (Fig. 3A).^40^ Similarly, neuromuscular exercise training was superior to usual exercise routine in 30 Iranian Military personnel for LBP intensity (SMD: −1.34; 95% CI: −2.14 to −0.54) (Fig. 2A), but there was no improvement in perceived disability scores (SMD: −0.73; 95% CI: −1.48 to 0.01) (Fig. 3A).^42^ Isolated lumbar extensor strengthening in Dutch Army personnel with LBP was not significantly better when compared to both wait-list and usual care (Fig. 3A).^35,36^ Postural exercises, when compared to no exercise, in 149 U.S. Army personnel with acute LBP demonstrated no benefit to long-term pain (SMD: 0.05; 95% CI: −0.35 to 0.45) or disability (SMD: −0.13; 95% CI: −0.53 to 0.27) (Figs. 2A and 3A).^32^ Progressive exercise programs developed for subacute LBP were not more beneficial compared to usual care for pain severity scores (SMD: 0.09; 95% CI: −0.35 to 0.52) (Fig. 2A).^45^

The use of oral glucosamine combination therapy for degenerative LBP showed no benefit over placebo for pain severity (SMD: −0.33; 95% CI: −0.91 to 0.26) (Fig. 2A) or perceived disability scores (SMD: −0.20; 95% CI: −0.78 to 0.38) (Fig. 3A).^37^ No improvement in LBP intensity scores were reported for electrotherapy modalities^38,45^ or adjunctive osteopathic manipulation^31^ (SMD: −0.50; 95% CI: −1.00 to 0.00) (Fig. 2A) when compared to usual care alone. In contrast, two studies reported positive outcomes for chiropractic manipulation in addition to usual care in 201 U.S. military personnel with acute LBP when compared to usual care alone.^34,46^ One study^46^ demonstrated reduced pain intensity (SMD: −0.69; 95% CI: −1.08 to −0.31) (Fig. 2A) and disability (SMD: −0.62; 95% CI: −1.00 to −0.24) (Fig. 3A) with adjunct chiropractic manipulation. The SMD could not be calculated for the other study because of inadequate available data.^34^

Early physiotherapy for acute LBP in active U.S. military personnel was beneficial in reducing pain intensity scores (SMD: −0.39; 95% CI: −0.75 to −0.02) (Fig. 2A) and pain disability scores (SMD: −0.45; 95% CI: −0.82 to −0.09) (Fig. 3A) when compared to usual care.^39^ Participation in a multidisciplinary pain program also demonstrated reductions in LBP disability scores (SMD: −0.86; 95% CI: −1.58 to −0.14) (Fig. 3A); however, improvement in pain intensity scores was not significant when compared to usual care (SMD: −0.30; 95% CI: −0.98 to 0.39) (Fig. 2A).^29^

#### Neck pain

The use of home cervical traction had no effect on reducing the intensity of flight-related neck pain compared to no traction in 21 Danish fighter pilots (SMD: −0.07; 95% CI: −0.97 to 0.73),^30^ nor did virtual reality self-kinematic training in 45 Israeli pilots (helicopter and fighter) compared to usual care (SMD: 0.36; 95%CI: −0.23 to 0.95) (Fig. 2B).^43^

#### Shoulder pain

There was no benefit of group exercise over usual physiotherapy care for perceived rotator cuff pain disability scores in 31 Canadian military personnel (SMD: 0.54; 95% CI: −0.18 to 1.26) (Fig. 3B).^41^

#### Knee pain

Electrotherapy modalities in isolation or in combination with exercise showed no benefit for knee pain severity compared to usual care (SMD: −0.17; 95% CI: −0.81 to 0.47) or exercise alone (SMD: −0.10; 95% CI −0.58 to 0.38 and SMD: −0.18; 95% CI: −0.66 to 0.30) (Fig. 2C).^44,47^ Similarly, the use of oral glucosamine combination therapy for degenerative joint disease did not produce significant improvement in knee pain scores (SMD: −0.41; 95% CI: −1.02 to 0.21) (Fig. 2C) or perceived disability compared to placebo in U.S. Navy personnel (SMD: −0.22; 95% CI: −0.83 to 0.39) (Fig. 3C).^37^

#### Chronic MSK pain

Participation in an interdisciplinary pain program reduced MSK pain intensity (SMD: −0.74; 95%CI: −1.24 to −0.24) and pain disability scores (SMD: −0.63; 95% CI: −1.12 to −0.12) in 66 U.S. military personnel (Figs. 2D and 3D).^33^

### Risk of Bias

Included studies were screened and assessed by two reviewers (C.L.B. and H.W.T.). There was a good level of agreement between the two reviewers (94% agreement; 89/95 judgments).^23^ All studies had a high risk of bias or some concerns. This was largely because of the methodological issues of potential unblinding or selective reporting. All studies included the use of patient-reported outcome measures, thus potentially influencing the results by knowledge of the intervention received. One study used a placebo-controlled intervention, hence reducing the risk of unblinding.^37^ Ten studies had reported preregistration with clinical trial repositories,^31,34,36,39,42–47^ of which three studies^36,39,46^ had published protocol papers.^48–50^ Twelve studies implemented an intention-to-treat analysis.^31,34–37,39,41,43–47^ Only eight studies reported acceptable levels of compliance.^29,32,33,35,39,40,42,46^

### Strength of the Evidence

The strength of evidence for the management of MSK pain in active serving military populations was “low” to “very low” using the GRADE framework. Despite all outcomes being assigned an *a priori* ranking of “high” for study design (RCT), initial rankings were double-downgraded because of the high risk of bias and imprecision of results. Some outcomes were further downgraded because of inconsistency in results of interventions.

## DISCUSSION

This review identified 19 relevant RCTs evaluating various conservative interventions to manage various MSK conditions in 1,408 active serving military personnel. The most frequently studied condition was LBP, followed by knee pain. The most common interventions were exercise therapy, manipulation therapy, and electrotherapy modalities. The relatively low number of studies for various MSK conditions and multiple interventions indicated that the available evidence is limited. Furthermore, the evidence was of “low” to “very low” strength, with some studies lacking methodological rigor and others deemed high risk or having concerns regarding the introduction of bias. Therefore, interpretation of the results and implications for patient management should be made with caution. The low quality of the evidence found in this review is in line with reviews of the management of MSK conditions in other populations.^51,52^ Similarly, a small body of evidence,^52^ heterogeneity of interventions and cohorts,^52^ and methodological rigor^51^ were cited as areas of potential bias. A disproportionate representation of certain body regions within the available research was also similar to reviews of the management of MSK pain in other populations.^51^ The most studied condition was LBP (13/19 studies). Although LBP is the most prevalent MSK disorder in active serving military groups,^53^ the prevalence of presentations varies depending on the service type and job role. For example, in army populations, lower limb injuries are the most prevalent.^54,55^ Almost 50% of all MSK injuries sustained over a 1-year period are attributable to the lower extremity,^55^ yet only three included studies investigated lower limb MSK conditions.^37,44,47^ Neck-related MSK pain is prevalent in pilots and aircrew,^56^ with an estimated incidence ranging from 29% to 57%.^56^ Moreover, neck- and shoulder-related MSK pain is common within the navy branch of service.^53^ Yet, there were only three identified RCTs that studied neck and neck-shoulder pain,^30,41,43^ which may limit the extrapolation of results to other prevalent MSK conditions.

Of the studies captured in the present review, exercise therapy was the most common form of intervention for the management of MSK pain (9/19 studies). Although the treatment effect varied, it appears exercise is better than rest. However, when interpreting the results of this review, it is important to consider the heterogeneous control conditions, allowing for participants to exercise as per “usual care.” The inclusion of exercise in the management of MSK pain is in line with high-quality clinical practice guidelines.^17^ Similarly, the implementation of multidisciplinary or interdisciplinary pain programs encompasses the consistent recommendations of using a patient-centered multimodal approach. The use of manipulative therapies is recommended as adjunct therapies as part of multimodal care, but not recommended as a stand-alone treatment.^17^

Despite attempting to capture a diverse range of MSK pain presentations and nonsurgical management strategies, only one study that met the inclusion criteria assessed a dietary supplementation intervention.^37^ Because of the potential for harm and implications for personnel’s deployability status, pharmacological interventions were also of interest. Unfortunately, we were unable to identify any RCTs evaluating the use of medication (prescription or over-the-counter) for the management of MSK pain. Rather, pain medication (e.g., NSAIDs and acetaminophen) were reported as a co-intervention in some studies.^31,34,37–40,45,46^ Recent literature on strategies for the management of MSK pain implemented by active serving personnel report that over-the-counter and opioid medications are frequently used.^12,14,57^ Thus, the lack of representation of pharmacological management strategies in this review may not be reflective of current practice.^14,57^ Future research comparing the effectiveness of these medications, including assessment of potential harms and benefits in this population, is required.

Factors to consider when assessing the effectiveness of an intervention include compliance, co-intervention, and contamination.^58^ Adherence to an intervention is crucial as it can greatly influence the perceived effectiveness of an intervention.^59^ Compliance is particularly relevant to exercise therapies and self-directed programs, both requiring participant motivation to complete the intervention protocol as intended.^60^ Less than half (42%) of the included studies reported acceptable levels of compliance,^29,32,33,35,39,40,42,46^ and no study controlled for co-interventions. Heterogeneous control conditions and minimal restriction on the use of therapies external to the study protocol may have impacted the overall treatment effect, e.g., variations in medications used, the types and intensity of physical activity, and rest. The use of pain education and advice regarding self-management strategies was commonly included in standard care protocols and may also have had an underestimated influence. For example, in general populations, education and self-management advice have been recommended as a first-line management approach for MSK pain.^17^ The application of intention-to-treat analysis by 63% of included studies would minimize the risk of bias introduced by poor compliance.

Inherent to studies assessing self-reported outcomes is the methodological difficulty of blinding the outcome assessor. In effect, the participant is the outcome assessor, and most nonpharmacological interventions cannot be delivered in a blinded manner to the participant. Another source of bias common to the included studies was being statistically underpowered. More than half^29–31,34–36,38–41,44,45^ of the included studies did not meet the *a priori* power calculations or desired sample size for pain-related outcomes, thus reducing the robustness of the findings. Many of the studies cited limitations to study recruitment in active serving populations. Reasons included the transient nature of posting cycles and impromptu taskings or deployments removing participants from the study setting. These limitations could be addressed by planning for longer time periods to capture data. Alternatively, ensuring data collection methods are transferrable between medical centers in order to avoid loss of follow-up.

The inclusion of only studies with an RCT design in active serving military populations narrowed the search yield to 19 studies. Studies were limited to active serving populations to capture the evidence for strategies applied in the unique structure of military medicine and the associated constraints of military service. Summarizing the evidence regarding the magnitude of treatment effect was complicated by variability in the included interventions and the control groups (placebo, wait-list, and active treatments). Many interventions were not performed in isolation or heterogeneous control conditions were used stating “usual care” or “standard care” as the comparison. These conditions may differ between studies depending on the setting, services involved, and country.

There are a number of additional limitations that must be considered when interpreting the findings of this review. The conclusions are limited because of the small number of studies and low strength of the evidence, and clinical heterogeneity precluded the inclusion of a meta-analysis. Additionally, the review itself was limited to English-language studies, potentially introducing language bias. An attempt to limit publication bias was made by conducting a search of the gray literature. Limiting the review to include only RCTs may also have excluded some high-quality observational studies within this population but attempted to provide the highest level of evidence available to military medicine. The majority of the included studies (13/19) were also based in U.S. military populations. The unequal representation of other countries further limits the generalizability of the evidence because of the inevitable variation in factors, such as medical systems, training, and equipment design.

To the authors’ knowledge, this is the first systematic review investigating the management of MSK conditions in active serving military populations. Subsequent reviews may benefit from broadening the scope to include high-quality observational studies to capture a wider range of interventions that are representative of current practice. Future high-quality, adequately powered trials are warranted. Producing a larger sample size and capacity to pool data would be beneficial to further validate these findings and enable evidence-based recommendations to guide military organizations and health care professionals.

## Supplementary Material

usac409_Supp

## Data Availability

No new data were generated or analyzed in support of this research.

## INSTITUTIONAL CLEARANCE

Institutional clearance does not apply.

## References

[1] Hainline B , Turner JA, Caneiro JP, et al: Pain in elite athletes—neurophysiological, biomechanical and psychosocial considerations: a narrative review. Br J Sports Med 2017; 51(17): 1259–64.doi: 10.1136/bjsports-2017-097890. 28827315

[2] Lovalekar M , Hauret K, Roy T, et al: Musculoskeletal injuries in military personnel-descriptive epidemiology, risk factor identification, and prevention. J Sci Med Sport 2021; 24(10): 963–9.doi: 10.1016/j.jsams.2021.03.016. 33824080

[3] Andersen KA , Grimshaw PN, Kelso RM, et al: Musculoskeletal lower limb injury risk in army populations. Sports Med Open 2016; 2(1): 22.doi: 10.1186/s40798-016-0046-z. PMC4851683 27213134

[4] Abt JP , Perlsweig K, Takashi N, et al: Effects of age and military service on strength and physiological characteristics of U.S. Army soldiers. Mil Med 2016; 181(2): 173–9.doi: 10.7205/MILMED-D-15-00036. 26837087

[5] Vallerand AH , Cosler P, Henningfield JE, et al: Pain management strategies and lessons from the military: a narrative review. Pain Res Manag 2015; 20(5): 261–8.doi: 10.1155/2015/196025. 26448972 PMC4596634

[6] Veterans Benefits Administration Annual Benefits Report Fiscal Year 2021: Compensation. Report VBA. Washington, DC: U.S. Department of Veteran Affairs. Available at https://www.benefits.va.gov/REPORTS/abr/docs/2021_compensation.pdf; June 2022 23, accessed June 23, 2022.

[7] Molloy JM , Pendergrass TL, Lee IE, Chervak MC, Hauret KG, Rhon DI: Musculoskeletal injuries and United States Army readiness Part I: overview of injuries and their strategic impact. Mil Med 2020; 185(9–10): e1461–7.doi: 10.1093/milmed/usaa027. 32175566

[8] Dijksma I , Bekkers M, Spek B, Lucas C, Stuiver M: Epidemiology and financial burden of musculoskeletal injuries as the leading health problem in the military. Mil Med 2020; 185(3–4): e480–6.doi: 10.1093/milmed/usz328. 31603239

[9] Armed Forces Health Surveillance Division Absolute and relative morbidity burdens attributable to various illnesses and injuries, active component: U.S. Armed Forces, 2021. MSMR 2022; 29(6): 2–9. 36250737

[10] Gun BK , Banaag A, Khan M, Koehlmoos TP: Prevalence and risk factors for musculoskeletal back injury among U.S. Army personnel. Mil Med 2022; 187(7–8): e814–20.doi: 10.1093/milmed/usab217. 34159385

[11] Reif S , Adams RS, Ritter GA, Williams TV, Larson MJ: Prevalence of pain diagnoses and burden of pain among active duty soldiers, FY2012. Mil Med 2018; 183(9–10): e330–7.doi: 10.1093/milmed/usx200. 29547946 PMC6115865

[12] Toblin RL , Quartana PJ, Riviere LA, et al: Chronic pain and opioid use in US soldiers after combat deployment. JAMA Intern Med 2014; 174(8): 1400–1.doi: 10.1001/jamainternmed.2014.2726. 24978399

[13] Athey A , and Overholser J: A systematic review of suicide risk in veterans: depression is a more powerful predictor than comorbid psychiatric disorders. Mil Behav Health 2018; 6(4): 1–10.doi: 10.1080/21635781.2018.1442757

[14] Sauers SE , Smith LB, Scofield DE, et al: Self-management of unreported musculoskeletal injuries in a U.S. Army brigade. Mil Med 2016; 181(9): 1075–80.doi: 10.7205/MILMED-D-15-00233. 27612356

[15] Heagerty R , Sharma J, Clayton J: A retrospective analysis of five years musculoskeletal injury data in British infantry recruits. Ann Musculoskelet Med 2017; 1(2): 032–8.doi: 10.17352/amm.000007.

[16] Babatunde OO , Jordan JL, Van der Windt DA, et al: Effective treatment options for musculoskeletal pain in primary care: a systematic overview of current evidence. PLoS One 2017; 12(6): e0178621.doi: 10.1371/journal.pone.0178621. PMC5480856 28640822

[17] Lin I , Wiles L, Waller R, et al: What does best practice care for musculoskeletal pain look like? Eleven consistent recommendations from high-quality clinical practice guidelines: systematic review. Br J Sports Med 2020; 54(2): 79–86.doi: 10.1136/bjsports-2018-099878. 30826805

[18] Harle CA , Danielson EC, Derman W, et al: Analgesic management of pain in elite athletes: a systematic review. Clin J Sport Med 2018; 28(5): 417–26.doi: 10.1097/JSM.0000000000000604. 30156573

[19] Sharp MA , Cohen BS, Boye MW, et al: U.S. Army physical demands study: identification and validation of the physically demanding tasks of combat arms occupations. J Science Med Sport 2017; 20(1): S62–7.doi: 10.1016/j.jsams.2017.09.013. 29054747

[20] Chou R , Atlas SJ, Stanos SP, et al: Nonsurgical interventional therapies for low back pain: a review of the evidence for an American Pain Society clinical practice guideline. Spine 2009; 34(10): 1078–93.doi: 10.1097/BRS.0b013e3181a103b1. 19363456

[21] Hughes JM , McKinnon CJ, Taylor KM, et al: Nonsteroidal anti‐inflammatory drug prescriptions are associated with increased stress fracture diagnosis in the US army population. J Bone Miner Res 2019; 34(3): 429–36.doi: 10.1002/jbmr.3616. 30352135 PMC6936225

[22] Moher D , Liberati A, Tetzlaff J, et al: Preferred reporting items for systematic reviews and meta-analyses: the PRISMA statement. BMJ 2009; 339: b2535.doi: 10.1136/bmj.b2535. PMC3090117 21603045

[23] Shea BJ , Reeves BC, Wells G, et al: AMSTAR 2: a critical appraisal tool for systematic reviews that include randomised or non-randomised studies of healthcare interventions, or both. BMJ 2017; 358: j4008.doi: 10.1136/bmj.j4008. PMC5833365 28935701

[24] Veritas Health Innovation : Covidence Systematic Review Software. Melbourne, Australia. 2022

[25] Higgins JP , Sterne JA, Savovic J, et al: A revised tool for assessing risk of bias in randomized trials. Cochrane Database Syst Rev 2016; 10(Suppl 1): 29–31.doi: 10.1002/14651858.CD201601.

[26] Guyatt GH , Oxman AD, Vist GE, et al: GRADE: an emerging consensus on rating quality of evidence and strength of recommendations. BMJ 2008; 336(7650): 924–6.doi: 10.1136/bmj.39489.470347.AD. 18436948 PMC2335261

[27] Deeks JJ Higgins JPT Altman DG : Chapter 10: Analysing data and undertaking meta-analyses. In: Deeks JJ, Higgins JPT and Altman DG, et al., eds. *Cochrane Handbook for Systematic Reviews of Interventions*. Version 6.2. Cochrane; 2021.doi: 10.1002/9781119536604.ch10.

[28] The Nordic Cochrane Centre : The Cochrane Collaboration Review Manager (ReVman), Version 5.4. Copenhagen, Denmark: The Nordic Cochrane Centre, 2020.

[29] Campello M , Ziemke G, Hiebert R, et al: Implementation of a multidisciplinary program for active duty personnel seeking care for low back pain in a U.S. Navy medical center: a feasibility study. Mil Med 2012; 177(9): 1075–80.doi: 10.7205/MILMED-D-12-00118. 23025138

[30] Chumbley EM , O’Hair N, Stolfi A, et al: Home cervical traction to reduce neck pain in fighter pilots. Aerosp Med Hum Perform 2016; 87(12): 1010–5.doi: 10.3357/AMHP.4625.2016. 28323586

[31] Cruser A , Maurer D, Hensel K, et al: A randomized, controlled trial of osteopathic manipulative treatment for acute low back pain in active duty military personnel. J Man Manip Ther 2012; 20(1): 5–15.doi: 10.1179/2042618611Y.0000000016. 23372389 PMC3267441

[32] Dettori JR , Bullock SH, Sutlive TG, et al: The effects of spinal flexion and extension exercises and their associated postures in patients with acute low back pain. Spine 1995; 20(21): 2303–12.doi: 10.1097/00007632-199511000-00008. 8553118

[33] Gatchel RJ , McGeary DD, Peterson A, et al: Preliminary findings of a randomized controlled trial of an interdisciplinary military pain program. Mil Med 2009; 174(3): 270–7.doi: 10.7205/MILMED-D-03-1607. 19354091

[34] Goertz CM , Long CR, Hondras MA, et al: Adding chiropractic manipulative therapy to standard medical care for patients with acute low back pain: results of a pragmatic randomized comparative effectiveness study. Spine 2013; 38(8): 627–34.doi: 10.1097/BRS.0b013e31827733e7. 23060056

[35] Harts CC , Helmhout PH, de Bie RA, et al: A high-intensity lumbar extensor strengthening program is little better than a low-intensity program or a waiting list control group for chronic low back pain: a randomised clinical trial. Aust J Physiother 2008; 54(1): 23–31.doi: 10.1016/S0004-9514(08)70062-X. 18298356

[36] Helmhout PH , Harts CC, Viechtbauer W, et al: Isolated lumbar extensor strengthening versus regular physical therapy in an army working population with nonacute low back pain: a randomized controlled trial. Arch Phys Med Rehabil 2008; 89(9): 1675–85.doi: 10.1016/j.apmr.2007.12.050. 18675396

[37] Leffler CT , Philippi AF, Leffler SG, et al: Glucosamine, chondroitin, and manganese ascorbate for degenerative joint disease of the knee or low back: a randomized, double-blind, placebo-controlled pilot study. Mil Med 1999; 164(2): 85–91.doi: 10.1093/milmed/164.2.85. 10050562

[38] Nayback-Beebe AM , Yoder LH, Goff BJ, et al: The effect of pulsed electromagnetic frequency therapy on health-related quality of life in military service members with chronic low back pain. Nurs Outlook 2017; 65(5): S26–33.doi: 10.1016/j.outlook.2017.07.012. 28893387

[39] Rhon DI , Miller RB, Fritz JM: Effectiveness and downstream healthcare utilization for patients that received early physical therapy versus usual care for low back pain: a randomized clinical trial. Spine 2018; 43(19): 1313–21.doi: 10.1097/BRS.0000000000002619. 29489568

[40] Brandt Y , Currier L, Plante TW, et al: A randomized controlled trial of core strengthening exercises in helicopter crewmembers with low back pain. Aerosp Med Hum Perform 2015; 86(10): 889–94.doi: 10.3357/AMHP.4245.2015. 26564676

[41] Ager AL , Roy JS, Gamache F, et al: The effectiveness of an upper extremity neuromuscular training program on the shoulder function of military members with a rotator cuff tendinopathy: a pilot randomized controlled trial. Mil Med 2019; 184(5–6): e385–93.doi: 10.1093/milmed/usy294. 30423137 PMC6525613

[42] Alvani E , Shirvani H, Shamsoddini A: Neuromuscular exercises on pain intensity, functional disability, proprioception, and balance of military personnel with chronic low back pain. J Can Chiropr Assoc 2021; 65(2): 193–206. 34658391 PMC8480371

[43] Bahat HS , German D, Palomo G, et al: Self-kinematic training for flight-associated neck pain: a randomized controlled trial. Aerosp Med Hum Perform 2020; 91(10): 790–7.doi: 10.3357/AMHP.5546.2020. 33187565

[44] Talbot LA , Solomon Z, Webb L, et al: Electrical stimulation therapies for active duty military with patellofemoral pain syndrome: a randomized trial. Mil Med 2020; 185(7–8): e963–71.doi: 10.1093/milmed/usaa037. 32248227

[45] Talbot LA , Webb L, Ramirez VJ, et al: Non-pharmacological home therapies for subacute low back pain in active duty military personnel: a randomized controlled trial. Mil Med 2021; usab382.doi: 10.1093/milmed/usab382. PMC8499864 34510214

[46] Vining R , Long CR, Minkalis A, et al: Effects of chiropractic care on strength, balance, and endurance in active-duty U.S. military personnel with low back pain: a randomized controlled trial. J Altern Complement Med 2020; 26(7): 592–601.doi: 10.1089/acm.2020.0107. 32543211

[47] Talbot LA , Brede E, Price MN, et al: Self-managed strength training for active duty military with a knee injury: a randomized controlled pilot trial. Mil Med 2019; 184(7–8): e174–83.doi: 10.1093/milmed/usy347. 30690578

[48] Rhon D , Fritz J: COMParative Early Treatment Effectiveness between physical therapy and usual care for low back pain (COMPETE): study protocol for a randomized controlled trial. Trials 2015; 16: 423.doi: 10.1186/s13063-015-0959-8. PMC4581511 26399603

[49] Vining R , Minkalis A, Long CR, et al: Assessment of chiropractic care on strength, balance, and endurance in active-duty U.S. military personnel with low back pain: a protocol for a randomized controlled trial. Trials 2018; 19(1): 671–671.doi: 10.1186/s13063-018-3041-5. 30518400 PMC6280458

[50] Helmhout PH , Harts CC, Staal JB, et al: Rationale and design of a multicenter randomized controlled trial on a ‘minimal intervention’ in Dutch army personnel with nonspecific low back pain. BMC Musculoskelet Disord 2004; 5(1): 40.doi: 10.1186/1471-2474-5-40. PMC533884 15535881

[51] Lin I , Wiles LK, Waller R, et al: Poor overall quality of clinical practice guidelines for musculoskeletal pain: a systematic review. Br J Sports Med 2018; 52(5): 337–43.doi: 10.1136/bjsports-2017-098375. 29175827

[52] Thornton JS , Caneiro JP, Hartvigsen J, et al: Treating low back pain in athletes: a systematic review with meta-analysis. Brit J Sports Med 2021; 55(12): 656–62.doi: 10.1136/bjsports-2020-102723. 33355180

[53] Morken T , Magerøy N, Moen BE: Physical activity is associated with a low prevalence of musculoskeletal disorders in the Royal Norwegian Navy: a cross sectional study. BMC Musculoskelet Disord 2007; 8(1): 56.doi: 10.1186/1471-2474-8-56. PMC1929072 17601352

[54] Davidson PL , Chalmers DJ, Wilson BD, et al: Lower limb injuries in New Zealand Defence Force personnel: descriptive epidemiology. Aust N Z J Public Health 2008; 32(2): 167–73.doi: 10.1111/j.1753-6405.2008.00195.x. 18412689

[55] Teyhen DS , Goffar SL, Shaffer SW, et al: Incidence of musculoskeletal injury in US Army unit types: a prospective cohort study. J Orthop Sports Phys Ther 2018; 48(10): 749–57.doi: 10.2519/jospt.2018.7979. 29787695

[56] Äng B , Harms-Ringdahl K: Neck pain and related disability in helicopter pilots: a survey of prevalence and risk factors. Aviat Space Environl Med 2006; 77(7): 713–9. 16856356

[57] Walker LA , Zambraski EJ, Williams RF: Widespread use of prescription nonsteroidal anti-inflammatory drugs among US Army active duty soldiers. Mil Med 2017; 182(3–4): e1709–12.doi: 10.7205/MILMED-D-16-00183. 28290947

[58] Krishna R , Maithreyi R, Surapaneni K: Research bias: a review for medical students. J Clin Diagn Res 2010; 4(2): 2320–4.

[59] Smyth EA , Newman P, Waddington G, et al: Injury prevention strategies specific to pre-elite athletes competing in Olympic and professional sports—a systematic review. J Sci Med Sport 2019; 22(8): 887–901.doi: 10.1016/j.jsams.2019.03.002. 30930142

[60] Teixeira PJ , Carraça EV, Markland D, et al: Exercise, physical activity, and self-determination theory: a systematic review. Int J Behav Nutr Phys Act 2012; 9(1): 78.doi: 10.1186/1479-5868-9-78. PMC3441783 22726453

