# Supplementary material for: Efficacy of Conservative Interventions for Musculoskeletal Conditions on Pain and Disability in Active Serving Military Personnel—A Systematic Review
Source: Mil Med. 2023 Jan 31;189(1-2):e66–75. doi: 10.1093/milmed/usac409 (PMC10824481; doi:10.1093/milmed/usac409)
Supplement: usac409_Supp [file usac409_supp.zip › MILMED-D-22-00310R2_Suppl Table S1.docx]

Supplementary Table 1. Characteristics of included studies and summary of results.

| Study (author, year) | Designs | Participants & condition | Interventions | Assessment points | Pain-related outcome measures | Results – outcomes in favour of intervention group | Summary of results |
| --- | --- | --- | --- | --- | --- | --- | --- |
| Ager et al (2019) | RCT | N=31 (intervention group =16; control group = 15) Canadian military personnel (Army >95%) with RC tendinopathy; male:female = 30:1 | 6 week group-supervised neuromuscular training program, 3 sessions/week.  *Control =* usual care | Baseline, 6 weeks & 12 weeks | DASH  WORC  NPRS | Mean change from baseline scores at end of 6 week program [mean (SD)]:   - DASH: Intervention group = -12.3 (10.2), control = -18.7 (12.7) - WORC: intervention group = 16.5 (14.0), control group = 30.7 (18.0)   NPRS mean change from baseline at end of 6 week program:   - Intervention group = 1.4 points, control group = 2.4 points | Parallel improvements in disability and pain over 6 week period for both groups  Clinically significant reduction in pain scores for usual care |
| Alvani et al (2021) | RCT | N=30 (intervention group = 15, control group = 15)  Iranian military personnel with chronic LBP, males only | 8 week neuromuscular exercise program 3 sessions/week, 60 minutes, in addition to usual physical activity & exercise routine  *Control =* usual exercise routine | Baseline & 8 weeks | VAS  ODI | Pre- & post-intervention scores [mean (SD)]   - VAS: intervention group = baseline 5.73 (1.63), 8 week 3.67 (1.96); control group = baseline 3.93 (1.75), 8 week 5.13 (1.29) - ODI: intervention group = baseline 16.80 (8.34), 8 week 9.87 (8.68); control group = baseline 13.87 (9.15), 8 week 17.20 (11.92) | Performing 8 weeks of neuromuscular exercises in addition to usual exercise routine reduced pain intensity and improved disability scores for chronic LBP |
| Bahat et al (2020) | RCT | N=45 (intervention group = 22, control group = 23)  Israeli Air Force personnel with neck pain, male:female = 41:4 | 4 week self-directed exercise program using neck VR system, 4/week, 5 minutes/session (20 minutes total/week) Completed in addition to usual care  *Control =* usual care | Baseline, 4 weeks & 6 months | VAS  NDI | Pre- & post- intervention scores [mean (SD)]   - VAS (/100mm): intervention group = baseline 36.4 (22.8), 8 week 25.7 (24.0); control group = baseline 49.5 (21.1), 8 week 26.9 (22.3) - NDI: intervention group = baseline 17.6 (8.9), median 15, 8 week median 10; control group = baseline 17.9 (10.4), median 16, 8 week median 10   Increased duration of exercise and improved NDI scores significant correlation (r=0.61, p=0.02) | No significant difference in pain intensity or disability between VR exercise program and usual care for neck pain  Increased duration of exercise positively influenced disability scores |
| Brandt et al (2015) | RCT | N=13 (intervention group = 6, control group = 7) US Air Force helicopter aircrew with LBP, males only | 5 core strength exercises on DVD (12reps each on any 4 days in a week over 12 week period)  *Control =* continuation of previous exercise regime | Baseline & 12 weeks | NPRS  GRCS  ODI | Comparing baseline to end of study period mean scores,   - NPRS flight score: intervention group -1.8 (p=0.034) and control group +0.7 (p=0.235) - ODI: intervention group -4.8 (p=0.026) and control group +1.7 (p=0.295)   End of the trial, mean GRCS in the intervention group was 4.0, compared to 0.0 in control group | Reduction in means NPRS (flight) and ODI in intervention group, with slight increase in control group  Mean daily NRPS scores demonstrated no difference between groups |
| Campello et al (2012) | RCT | N=33 (intervention group = 16, control group = 17) US Navy personnel with LBP (<12week history), male:female = 30:3 | 4 week multidisciplinary program up to 3 hours/day, 3 days/week.  *Control* = usual care. | Baseline, 4 weeks (post-intervention), & 12 weeks. | NPRS  ODI | Difference in mean scores at end of intervention period,   - Pain: 4 weeks 1.3 (p=0.074), 12 weeks 1.1 (p=0.310) - ODI: 4 weeks 10.3 (p=0.014*), 12 weeks 7.6 (p=0.117)   *significant improvement in ODI at end of intervention period. | Intervention group reported significantly lower perceived disability and less pain compared to control group at the end of the intervention period |
| Chumbley et al (2016) | RCT – Crossover design | N=21, Danish Air Force fighter pilots, neck pain, males only | 6 week home cervical traction device 10 minutes 3x/week. *Control phase* = no traction. | Pain: Daily – pre- & post-flying, & post-traction.  ROM: baseline, washout & completion final phase | NPRS | Initial pain levels on days with traction = NPRS 1.3, compared to days following traction = NPRS 1.1. Difference = 0.2, small but found to be significant (p=0.014)  Significant reduction in pain levels post-traction on flying days NPRS -0.3 (0.3) (p<0.05)    Correlation between pain levels and benefit from traction use demonstrated linear relationship (Spearman’s coefficient -0.729, DF=10, p=0.007) | Pain levels increased post-flight, and post-traction pain levels reduced in a linear fashion relative to reported pain post-flight  The difference in pain levels after traction compared to initial levels were not significant |
| Cruser et al (2012) | RCT | N=63 (intervention group = 33, control group = 30) US military personnel, Acute LBP, male:female = 33:27 *data analysed | Osteopathic manipulation 1x/week for 4 weeks  *Control* = usual care | Baseline, 1 week after treatment 1-3 & 4 weeks post final treatment | Quadruple VAS  RMDQ | Difference of means from baseline to end-point (95%CI),   - “Pain now” -1.47 (-2.75, -0.18) (p=0.026)   Significant difference for time to improvement for “pain at best,” intervention group reporting 30% improvement (MCID) at an earlier session than control group (p=0.004) | Intervention group showed significantly greater improvements in “pain now” from baseline to end-point  Significant effect of time on improvement in pain and functioning - both groups improving on average over time |
| Dettori et al (1995) | RCT – 5-arms (4 intervention groups) | N=149 (intervention group = 119, control group = 30) US Army personnel, acute LBP, male:female = 120:29 | Postural direction-based exercise program. 20 minutes ice application followed by prescribed posture and movement-based exercises, 3x/day  *Control* = ice only, no exercise program | Baseline, week 1, 2, 4 & 8 study period; & 6-12 months post-study | NPRS  RMDQ | After 1 week, intervention groups reported greater improvements than control group in:   - RMDQ (mean, 95%CI): Intervention 7.35 (6.77, 11.23); control 9.0 (6.40, 8.30) (p=0.054) - Return to work: intervention 18.3%; control 3.6% (p=0.053) | Flexion and extension exercise groups did not differ in any outcome over the 8 week intervention period  After 1 week, intervention groups reported reduced disability scores and higher proportion returning to work compared to the control group  There was no difference in recurrence of LBP after 6-12 months between groups |
| Gatchel et al (2009) | RCT | N=66 (intervention group = 30, control group = 36) US military personnel, chronic musculoskeletal pain, male:female = 44:22 | Interdisciplinary pain management program  *Control* = usual care (relative to anaesthesia pain clinic) | Baseline, post-intervention, 6 months & 1 year | VAS  MPI  ODI  DPDQ | Post-treatment differences group comparisons (post-treatment score [mean, (SD)],   - VAS: intervention 3.8 (2.3), control 6.0 (2.1) (p=0.008) - DPDQ: intervention 54.3 (21.8), control 76.6 (21.2) (p=0.003) - ODI: intervention 11.0 (5.4), control 17.8 (4.5) (p=0.002)   6month follow up outcome results for 2 groups [mean (SD)],   - VAS: intervention 4.0 (2.3), control 6.6 (2.0) (p=0.002) - DPDQ: intervention 51.3 (24.3), control 81.7 (19.7) (p<0.001) - ODI: intervention 10.3 (7.7), control 19.5 (5.5) (p<0.001)   1 year follow-up outcome results for 2 groups (mean),   - VAS: intervention 3.0, control 5.0 (p=0.05) | Intervention group participants reported significantly greater improvements in self-reported pain, disability, functional status and fitness for military duty post-treatment and follow-up points compared to standard care group |
| Goertz et al (2013) | RCT | N=91 (intervention group = 45, control group = 46) US military personnel, acute LBP, male:female = 78:13 | Usual care plus chiropractic manipulative therapy, up to 2 visits/week for 4 weeks *Control =* usual care | Baseline, 2 weeks & 4 weeks | NPRS  RMDQ | Adjusted mean differences between groups at 2 weeks (95% CI),   - RMDQ: 3.9 (1.8, 6.1) (p<0.001) - NPRS: 2.2 (1.2, 3.1) (p<0.001)   At 4 weeks (95% CI),   - RMDQ: 4.0 (1.3, 6.7) (p=0.0014) - NPRS: 1.2 (0.2, 2.3) (p=0.02) | Mean scores for RMDQ decreased for both groups during the study period, but mean scores for RMDQ and NPRS were significantly better in the intervention group |
| Harts et al (2008) | RCT – 3-arms with control group randomised after initial treatment period. | N=65 (intervention groups = 44, control group = 21) Dutch Army personnel, chronic non-specific LBP, males only | 8 week lumbar extensor strength program – High intensity (progressive starting 50%MVC) and low intensity (non-progressive 20%MVC) groups  *Control* = regular physiotherapy, no back extensor strengthening | Baseline, 8 weeks, 24 weeks | GPE  RMDQ | GPE mean (95% CI) difference between high intensity and wait-list control groups at 8weeks post-intervention = 39% (14%, 64%). | Self-assessed improvement in back symptoms was reported to be on average 39% greater in high-intensity group compared to waiting list control at 8 weeks  No other differences between groups for any outcome at 8weeks or 24 weeks was observed |
| Helmhout et al (2008) | RCT | N=127 (intervention group = 71, control group = 56) Dutch Army personnel, LBP, male:female = 123:4 | 10 week lumbar extensor strength training 2 sessions/week  *Control* = regular physiotherapy, no back extensor strengthening | Baseline, 5 weeks, 10 weeks, 6 months & 12 months. | GPE  RMDQ  PSFS | No significant differences between groups at any time point reported | Both groups showed improvements in outcomes over time, with short term improvements remaining stable or slightly increased towards the 12 month follow-up  No significant differences between the 2 groups were observed for any outcome measure at any time |
| Leffler et al (1999) | RCT – Placebo-controlled crossover design | N=34, US Navy, chronic knee and/or LBP, males only | 8 week use of placebo capsule or Cosamin (chondroitin sulfate & manganese ascorbate) 3 divided doses  *Control phase* = continued normal routines including exercise. Not permitted to take NSAIDs but able to take acetaminophen | Average of 2 measures in baseline period & final 2 weeks of 8 week treatment phases | VAS  RMDQ  Lequesne Index | Overall summary results, statistically significant improvement in pain VAS (p=0.02)  When separated, most improvement attributable to knee data. Mean change pain VAS (95% CI): In clinic -26.6% (-53.0%, -0.2%). Diary entries -28.6% (-52.7%, -4.5%) | Knee osteoarthritis symptoms were reduced, but no change to functional measures were observed  Benefits for LBP was not demonstrated |
| Nayback-Beebe et al (2017) | RCT | N=75 (intervention group = 36, control group 32 *baseline measures reported) US military personnel, chronic LBP, male:female = 52:23 | 4 week of usual care plus self-administered PEMF, 30minutes 3x/week  *Control* = usual care | Baseline, 4 weeks & 8 weeks | NPRS | Pain NPRS at baseline [mean (SD)],   - Intervention: 4.3 (1.8) - Control: 3.5 (2.3)   4 weeks,   - Intervention: 3.9 (1.6) - Control: 3.8 (2.0)   8 weeks,   - Intervention: 3.8 (2.3) - Control: 3.4 (2.2) | Intervention group demonstrated no significant changes to pain symptoms and severity compared to the control group at any timepoint |
| Rhon et al (2018) | RCT | N=119 (intervention group = 58, control group = 61) US military personnel, LBP, male:female = 101:18 | Early physical therapy commenced within 72 hours. Up to 8 sessions  *Control* = usual care (education, medication, limited duty restrictions) | Baseline, 4 weeks, 3 months & 1 year. | NPRS  ODI  GRCS | Between group mean difference (SD, 95% CI) at 4 weeks for ODI 4.38 (25.95, 0.41 to 10.10) (p=0.042)  Mean (SD, 95%CI) for ODI at 4 weeks,   - Intervention: 22.19 (12.80, 18.83 to 25.55) - Control: 26.57 (12.29, 23.43, 29.71) | No between group difference in ODI is noted after 1 year  Between group difference is seen after 4weeks in favour of early physical therapy intervention compared to usual care  No difference between groups in adjusted NPRS scores at any timepoint |
| Talbot et al (2019) | RCT | N=78 (WALK group = 20, NMES group = 19, combination group = 20, control group = 19) US military personnel with knee pain, male:female = 6:7 | 18 week program in addition to usual care  WALK = graduated strength walking program, minimum 30minutes 4days/week, weight vest added at week 7  NMES = 4x 15 minute sessions/week using NMES on knee extensors  Combination = 4x 15 minute NMES sessions/week, graduated walking program as per WALK group  *Control =* usual care | Baseline, 6 weeks, 12 weeks & 18 weeks | VAS | Pre- & post-intervention pain severity scores [mean (SD)]:   - WALK: baseline 5.7 (1.7), 18 weeks 4.3 (2.4) - NMES: baseline 4.9 (2.1), 18 weeks 3.6 (2.4) - Combination: baseline 5.1 (1.9), 18 weeks 3.9 (2.3) - Control: baseline 5.4 (1.9), 18 weeks 5.3 (2.6) | Pain severity reduced over the course of the study, but no significant difference between groups for pain severity scores |
| Talbot et al (2020) | RCT | N=130 (NMES group = 33, TENS group = 33, combination group = 30, control group = 34)  US military personnel with anterior knee pain, male:female = 101:29 | 9 week program in addition to prescribed home exercise program (HEP)  NMES = 20 minutes stimulation whilst performing daily HEP on alternate days, graduated intensity  TENS = 20 minutes stimulation whilst performing daily HEP on alternate days  Combination = NMES and TENS performed as per above protocols on alternating days  *Control =* HEP only | Baseline, 3 weeks, 6 weeks & 9 weeks | VAS | Pre – & post-intervention pain severity scores [mean (SD)]:   - NMES: baseline 3.42 (1.66), 9 weeks 2.44 (1.79) - TENS: baseline 4.24 (1.76), 9 weeks 3.01 (2.33) - Combination: baseline 3.98 (1.77), 9 weeks 3.27 (2.26) - Control: baseline 3.48 (1.84), 9 weeks 2.77 (2.22) | Pain severity reduced in all groups, there were no statistically significant differences in reported resting pain between groups |
| Talbot et al (2021) | RCT | N=128 (NMES group = 43, PEP group = 42, control group = 43)  US military personnel with subacute LBP, male:female = 106:22 | 9 week program in addition to primary care management  NMES = 30 minutes stimulation/day alternating every other day between lumbar & abdominal sites  PEP = progressive exercise program, 60 minute sessions/every second day; 3x 3 week phases  *Control =* primary care management, weekly communication | Baseline, 3 weeks, 6 weeks & 9 weeks | VAS | Pre- & post-intervention pain severity scores [mean (SD)]:   - NMES: baseline 4.57 (2.2), 9 weeks 4.07 (3.1) - PEP: baseline 4.78 (2.0), 9 weeks 3.93 (2.7) - Control: baseline 4.43 (2.0), 9 weeks 3.28 (2.6) | Current pain severity scores reduced in all groups, no significant between group difference were noted (p=0.57) |
| Vining et al (2020) | RCT | N=110 (intervention group = 55, control group = 55)  US military personnel with LBP, male:female = 91:19 | 4 weeks of chiropractic care/spinal manipulation – number of visits determined case by case  *Control =* wait-list control, able to seek other healthcare except spinal manipulation/chiropractic care | Baseline & 4 weeks | NPRS  RMDQ | Mean change (95%CI) from baseline to post-intervention:   - RMDQ: intervention group -3.2 (-4.4, -2.2), control group -1.0 (-2.0, -0.1) - NPRS: Intervention group -0.9 (-1.3, -0.6), control group 0 (-0.4, 0.3)   Difference in mean change between groups (95%CI):   - RMDQ: -2.1 (-6.5, -0.8) - NPRS: -1.0 (-1.5, -0.4) | Short-term improvements in LBP severity and disability with chiropractic care compared to wait-list controls |

RC = rotator cuff; LBP = low back pain; RCT = randomised control trial; SD = standard deviation; CI = confidence interval; US = United States; NSAIDs = non-steroidal anti-inflammatories; PEMF = pulsed electromagnetic frequency; NMES = neuromuscular electrical stimulation; TENS = transcutaneous electrical nerve stimulation; HEP = home exercise program; MVC = maximal voluntary contraction; VAS = visual analogue scale; NPRS = numerical pain rating scale; ODI = Oswestry disability index; RMDQ = Roland-Morris disability questionnaire; MPI = multidimensional pain inventory; NDI = Neck Disability Index; PSFS = patient specific functional scale; GRCS = global rating of change scale; GPE = global perceived effect; WORC = Western Ontario rotator cuff questionnaire; DASH = Disabilities of the arm, shoulder and hand questionnaire; DPDQ = Dallas Pain & Disability Questionnaire; MCID = minimal clinically important difference; VR = virtual reality.
